# Comparative assessment of fully laparoscopic Duhamel-Z with minimal rectorectal dissection vs. laparoscopy-assisted Duhamel-Z with blunt manual rectorectal dissection for total colonic aganglionosis

**DOI:** 10.3389/fped.2023.1255899

**Published:** 2023-10-04

**Authors:** Go Miyano, Hisae Iida, Yu Ebata, Eri Abe, Haruki Kato, Takafumi Mikami, Junya Ishii, Geoffrey J. Lane, Atsuyuki Yamataka, Tadaharu Okazaki

**Affiliations:** ^1^ Pediatric Surgery, Juntendo University Urayasu Hospital, Chiba, Japan; ^2^ Pediatric General & Urogenital Surgery, Juntendo University School of Medicine, Chiba, Japan

**Keywords:** total colonic aganlionosis, duhamel-Z, laparoscopy, retrorectal dissection, continence

## Abstract

**Aims:**

Early postoperative outcome (EPO) was compared between fully laparoscopic Duhamel-Z (F-Dz) and laparoscopy-assisted Duhamel-Z (A-Dz) anastomoses performed for total colonic aganglionosis (TCA).

**Methods:**

EPO was assessed quarterly for the first year after F-Dz/A-Dz using a continence evaluation score (CES) based on stool frequency (motions/day) and stool consistency (0 = liquid, 1 = soft, 2 = formed), presence of anal erosion (0 = severe, 1 = moderate, 2 = mild), and incidence of enterocolitis.

Surgical technique involved taking the ileostomy down, dissecting the colon laparoscopically, and preparing the pull-through ileum through the stoma wound. In F-Dz (*n* = 3), a working port (SILS trocar) was inserted, and laparoscopic retrorectal dissection with forceps used to create a retrorectal tunnel from the peritoneal reflection extending downward as narrow as possible along the posterior wall of the rectum to prevent lateral nerve injury and preserve vascularity. After completing the tunnel, the ileum was pulled-through from an incision on the anorectal line and a *Z*-shaped ileorectal side-to-side anastomosis performed without a blind pouch. In A-Dz (*n* = 11), the retrorectal pull-through route was created through a Pfannenstiel incision using blunt manual (finger) dissection along the anterior surface of the sacrum.

**Results:**

Subject backgrounds were similar. Mean quarterly data were: frequency (F-Dz: 4.67, 4.67, 4.67, 3.33) vs. (A-Dz: 7.27, 7.09, 6.18, 5.36) *p *< .05; consistency (F-Dz: 0.33, 0.67, 0.67, 0.67) vs. (A-Dz: 0.27, 0.45, 0.70, 0.73) *p *= ns; anal erosion (F-Dz: 0.33, 0.33, 0.33, 0.67) vs. (A-Dz: 0.18, 0.36, 0.45, 0.64) *p *= ns; and enterocolitis (F-Dz: 1 episode in 1/3 cases or 33.3%) vs. (A-Dz: 7 episodes in 6/11 cases or 54.5%) *p *= ns.

**Conclusions:**

Overall, EPO after F-Dz was better than after A-Dz.

## Introduction

Surgical intervention for total colonic aganglionosis (TCA) has been reported and modified over the years (1–4) without consensus for a treatment of choice being reached. The Duhamel procedure gained favor because there was less pelvic dissection and a segment of aganglionic rectum was used as a reservoir to reduce the frequency of defecation, improving bowel continence. Ikeda (5) modified the Duhamel procedure by introducing a *Z*-shaped ileorectal side-to-side anastomosis in 1967 that eliminated the rectal reservoir and dividing the colorectal septum, completely. In 2017, the first author reported gradual improvement in postoperative bowel continence in TCA patients treated by laparoscopy-assisted Duhamel-Z anastomosis (A-Dz) over time using a comprehensive continence evaluation score (CES) (6).

Here, early postoperative outcome (EPO determined from selected CES criteria considered most likely to influence quality of life such as frequency/consistency of motions, presence of anal erosion, and incidence of enterocolitis) after fully laparoscopic Duhamel-Z anastomosis (F-Dz) and A-Dz were compared in TCA patients.

## Methods

A-Dz was performed from 2009 to 2019 and F-Dz since 2020. Cases in this series were consecutive. Surgically, for both F-Dz and A-Dz, four 5-mm ports were used to dissect the entire colon beginning from the peritoneal reflection at the sigmoid colon progressing proximally to the ileostomy site following the bowel wall closely using conventional laparoscopic techniques. After the ileostomy was taken down under laparoscopic control (7), pull-through ileum was prepared by dissecting the ileal mesentery through the abdominal ileostomy wound.

**Figure 1:**
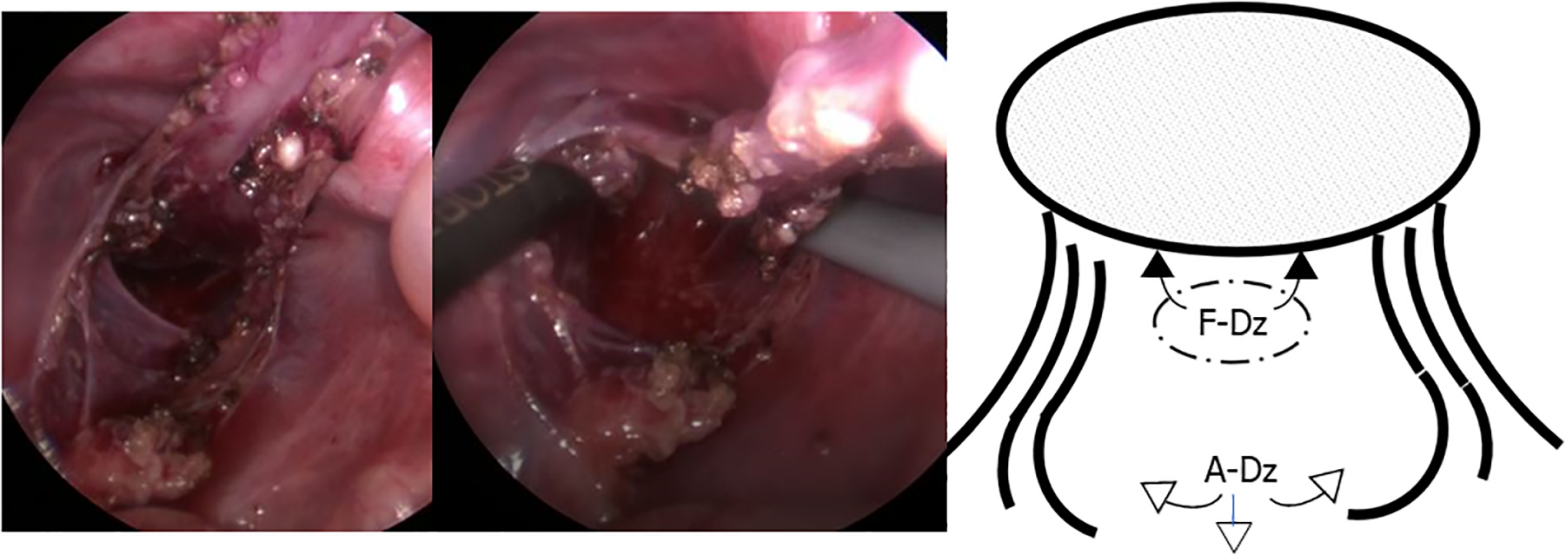
Laparoscopic retrorectal dissection. Retrorectal dissection was essentially performed bluntly and as narrowly as possible, along the posterior wall of the rectum to prevent lateral nerve injury and preserve lateral vascularity in F-Dz. F-Dz: fully laparoscopic Duhamel-Z, A-Dz: laparoscopy-assisted Duhamel-Z.

**Figure 2:**
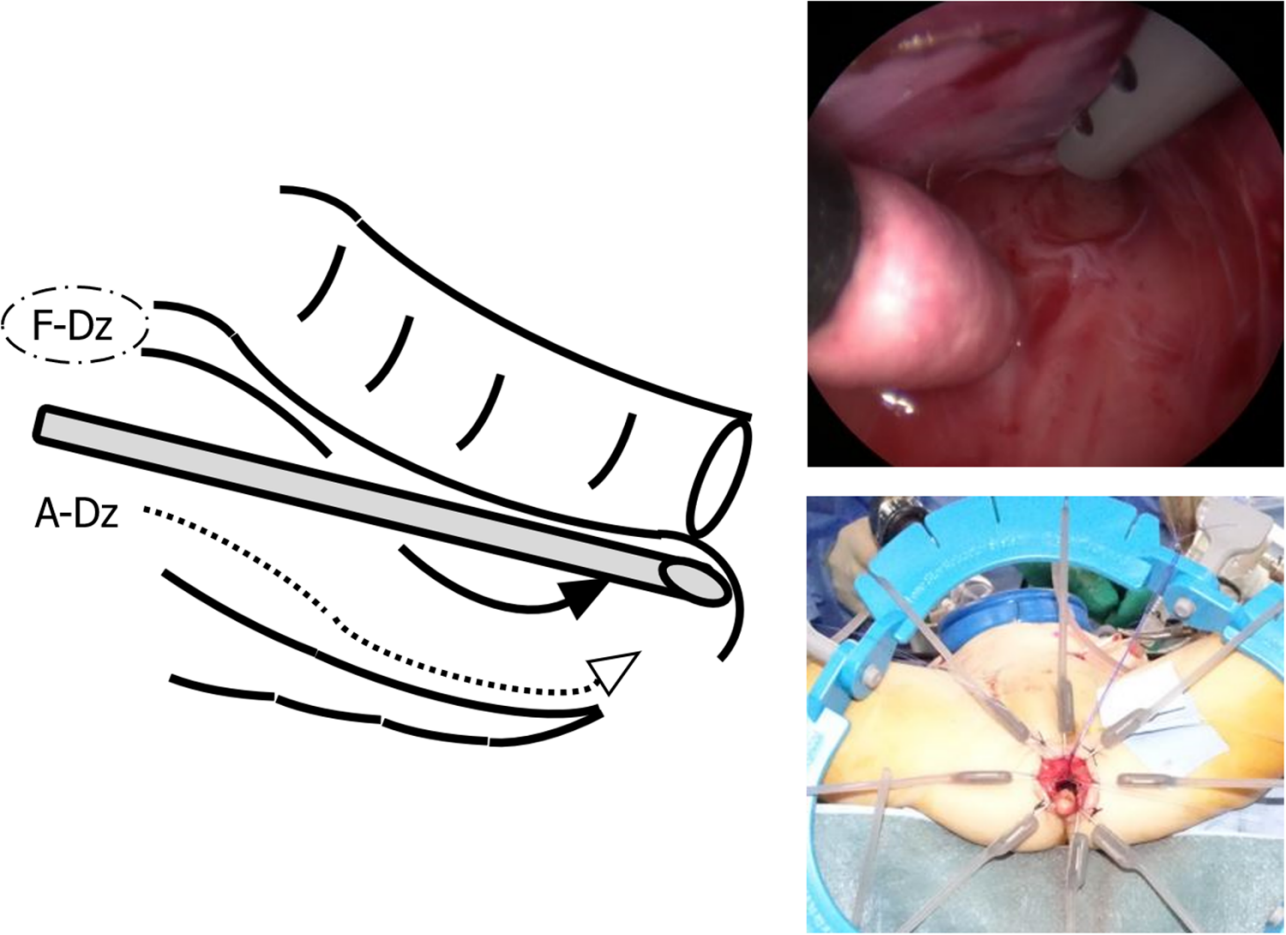
Laparoscopic retrorectal dissection reaching the anorectal line. When laparoscopic dissection/tunneling reached the anorectal line, the ileum was pulled-through through the narrow retrorectal tunnel without additional dissection in F-Dz taking care to protect the surgical anal canal. In A-Dz, an extensive retrorectal dissection along the anterior surface of the sacrum (arrowheads) was required.

**Figure 3:**
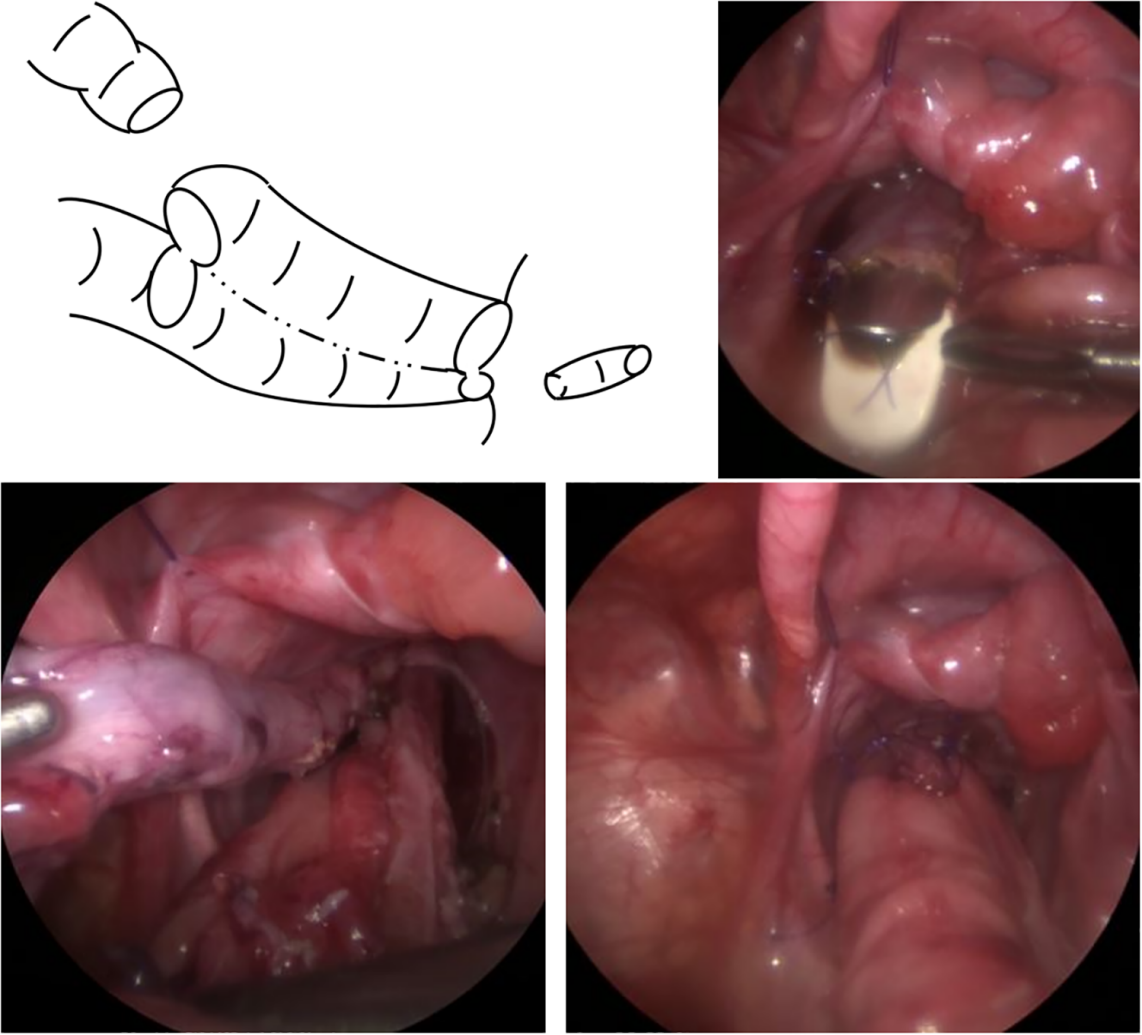
Z-shaped ileorectal side-to-side anastomosis without a blind reservoir. The incised posterior wall of the rectum and pulled-through ileum were anastomosed at the anus, and a surgical stapler used to divide the posterior rectal wall and anterior ileal wall. Finally, the anterior wall of the upper rectum and the upper edge of the incised anterior wall of the normal ileum were anastomosed to complete the Z-shaped ileorectal side-to-side anastomosis without a blind reservoir.

For F-Dz, a Free Access^TM^ working port/platform (Top Corporation, Tokyo, Japan) was placed in the abdominal wound. Laparoscopic retrorectal dissection was performed using forceps extending downward to create a retrorectal tunnel. From the peritoneal reflection, dissection was performed as close as possible to the posterior wall of the rectum to prevent lateral nerve injury and preserve lateral vascularity (Figure 1). When laparoscopic dissection/tunneling reached the anorectal line (ARL), an incision was made on the ARL taking care to protect the surgical anal canal, and the ileum was pulled-through from this incision through the narrow retrorectal tunnel without additional dissection (Figure 2). The rectal stump was resected 2 cm above the peritoneal reflection, and a transverse incision was made on the anterior wall of the ganglionic ileum at the level of the proximal rectal end. The posterior wall of the upper rectum and lower edge of the incised anterior wall of the ileum were then anastomosed very tightly using interrupted sutures. The posterior wall of the rectum was incised on the ARL and the pulled-through ileum was anastomosed to the anus circumferentially in a single layer using interrupted 5-0 monofilament sutures. A 60 mm long Tri-Staple^TM^ technology surgical stapler was inserted through the anus to divide the posterior rectal wall and anterior ileal wall. Finally, the anterior wall of the upper rectum and the upper edge of the incised anterior wall of the normal ileum were anastomosed in two layers to complete the Z-shaped ileorectal side-to-side anastomosis without a blind pouch (Figure 3).

For A-Dz, after the ileostomy was taken down, a Pfannennstiel incision was used to create an extensive retrorectal pull-through route using finger dissection along the anterior surface of the sacrum and perform a Z-shaped ileorectal side-to-side anastomosis as an open procedure. A-Dz was then completed using the same technique described for F-Dz.

Data were collected for demographics, clinical presentation, presence of associated anomalies, surgical management, and perioperative complications. EPO assessment was performed quarterly for one year by specialist nursing staff with wound, ostomy, and continence (WOC) care certification; CES was determined from frequency (motions per day) and consistency (scored using: 0 = watery, 1 = soft, and 2 = formed, to calculate a consistency score) data by WOC staff; severity of anal erosion determined by WOC staff using: 0 = severe (requiring constant corticosteroid administration), 1 = moderate (requiring intermittent corticosteroid administration), 2 = mild (requiring emollients only); and the incidence of enterocolitis. Data were compared between F-Dz (*n* = 3) and A-Dz (*n* = 11). Data for A-Dz were obtained from a previously published report by the same authors.

Data were analyzed using standard statistical methods with the software Statcel-2 (OMS Publishing Inc., Saitama, Japan). Demographics were compared using Bonferroni corrected *post hoc* tests. EPO determined from selected CES criteria were compared using the Student's *t*-test. For all statistical tests, *p *< .05 was used to determine significance. Methodology and ethics were in accordance with the Helsinki Declaration (2013).

## Results

F-Dz cases were 2 females and 1 male; A-Dz cases were 5 females and 6 males. Mean age and mean weight were 7.6 months old and 7.1 kg, respectively in F-Dz and 10.2 months and 8.4 kg in A-Dz; differences were not statistically significant (*p *= ns for age; *p *= ns for weight). Mean lengths of aganglionic ileum resected (24.0 cm in F-Dz vs. 19.5 cm in A-Dz) were statistically similar (*p *= ns). Mean operative times were 5.9 h in F-Dz vs. 6.2 h in A-Dz; differences were not statistically significant (*p *= ns). There were no perioperative complications in either group.

**Figure 4:**
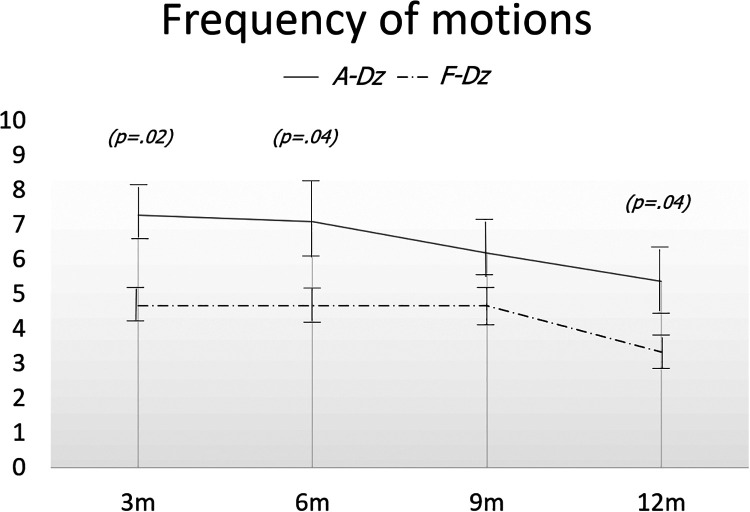
Frequency of stools in both groups. Frequency of stools was significantly lower in F-Dz than in A-Dz at 3, 6, and 12 months postoperatively.

**Figure 5:**
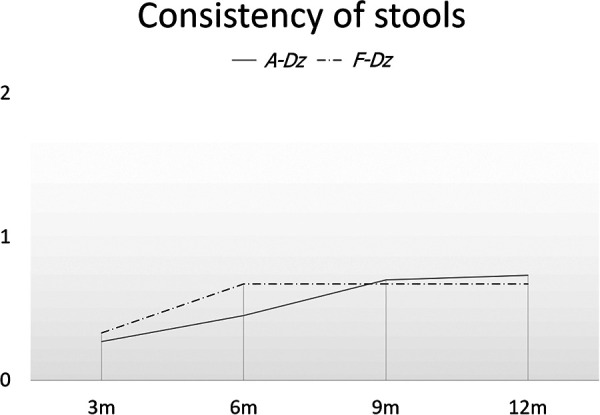
Consistency of stools in both groups. Consistency of stools was not statistically different between F-Dz and A-Dz.

**Figure 6:**
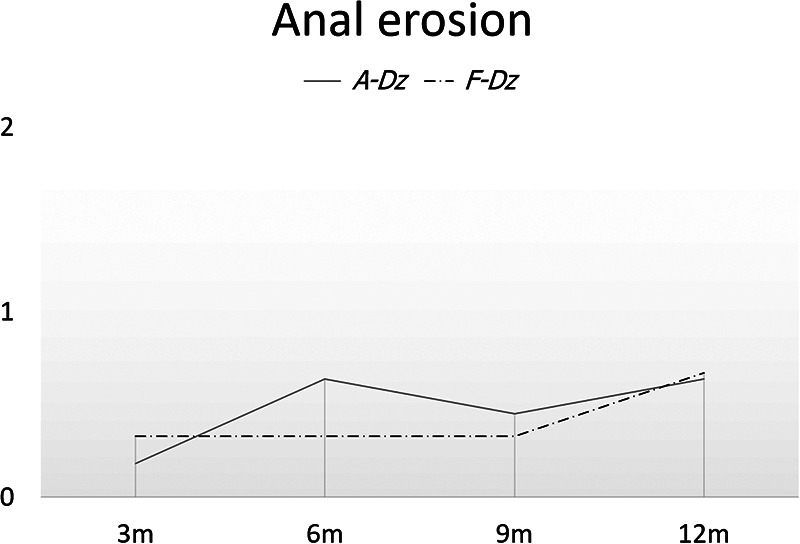
Severity of anal erosion in both groups. Severity of anal erosion was not significantly different between F-Dz and A-Dz.

No subjects were constipated during the immediate postoperative period. Mean frequencies of stools/day at 3, 6, 9 and 12 months after F-Dz were: 4.67 ± 0.5, 4.67 ± 01.1, 4.67 ± 0.5, and 3.33 ± 0.5, respectively; after A-Dz were: 7.27 ± 1.4, 7.09 ± 1.6, 6.18 ± 1.3, and 5.36 ± 1.4, respectively; *p *< .05 at 3, 6, and 12 months (Figure 4). Mean consistency scores at 3, 6, 9, and 12 months after F-Dz were: 0.33 ± 0.5, 0.67 ± 0.5, 0.67 ± 0.5, and 0.67 ± 0.5, respectively; after A-Dz were: 0.27 ± 0.4, 0.45 ± 0.5, 0.70 ± 0.6, and 0.73 ± 0.6, respectively; *p *= ns throughout the study period (Figure 5). Mean erosion scores at 3, 6, 9, and 12 months after F-Dz were: 0.33 ± 0.5, 0.33 ± 0.5, 0.33 ± 0.5, and 0.67 ± 0.5, respectively; after A-Dz were: 0.18 ± 0.4, 0.36 ± 0.5, 0.45 ± 0.5, and 0.64 ± 0.5, respectively; *p *= ns throughout the study period (Figure 6). Enterocolitis occurred in 1/3 F-Dz cases (33.3%) and there were 7 episodes in 6/11 A-DZ cases (54.5%); *p *= ns.

## Discussion

Duhamel-Z is indicated primarily for rectosigmoid Hirschsprung disease. When performed as an open procedure, retrorectal dissection is performed transanally, using the fingers through a transverse incision on the posterior rectal wall to extend full-thickness upward along the surface of the sacrum, followed by creating a wide retrorectal tunnel between the anal side and the peritoneal cavity (8). However, when performed laparoscopically for TCA, retrorectal dissection is performed laparoscopically. A report detailing transanal retrorectal dissection during laparoscopic Duhamel-Z with the direction of dissection extending upward from a full-thickness hemicircumferential incision on the posterior wall of the rectum required the retrorectal tunnel to be wide enough to allow the folded rectum to be grasped and pulled-down through it (9). Thus, a feature of F-Dz used in the current report is that local trauma during dissection was minimized and lateral rectal ligaments preserved by using peanut gauze swabs for dissection in view of the density of neurovascular and venous plexuses on the pelvic surface of the sacrum. Similarly, electrocautery was not used for hemostasis to prevent compromising the rectal blood supply and presacral innervation (10) that could potentially disrupt postoperative bowel function.

F-Dz was also considered less invasive because laparoscopy ensured more precise and meticulous dissection of the posterior wall, preventing more extensive dissection of the sacrum associated with manual (finger) dissection or open dissection during A-Dz. As a result, better control of defecation reflected by the lower frequency of stools after F-Dz may be related to minimizing injury associated with manual dissection of the sacral surface or preventing the creation of a narrow pull-through route by limited dissection. The relatively younger age of F-Dz patients may be implicated in stool frequency results although age differences between groups in this study were not statistically significant. Since publishing the report from which A-Dz data was obtained, longer follow-up has shown further gradual improvement in overall CES over time, emphasizing that the anorectum has reserve for functional resolution after surgical intervention.

Major limitations of this study are the small sample size and short duration of follow-up. The F-Dz group had surgery at a relatively younger but not statistically significant age which could influence postoperative fecal continence, an issue that could not be confirmed because of the small series and short follow-up. However, despite the small sample size and short follow-up period for F-Dz in this study, improvement in EPO after F-Dz was considered worth reporting for its potential for enhancing the treatment of TCA and improving the quality of life of postoperative TCA patients. Since extending the follow-up period and increasing the sample size will allow a more comprehensive evaluation of surgery in the long-term, outcome after F-Dz is being closely monitored and will be reported when sufficient data is available.

## Data Availability

The raw data supporting the conclusions of this article will be made available by the authors, without undue reservation.

## Supplementary material

SUPPLEMENTARY VIDEO 1

Retrorectal dissection toward the perineum. Retrorectal dissection was performed as close as possible to the posterior wall of the rectum, and when dissection/tunneling reached the anorectal line, the laparoscopic surgeon can see the ARL being pressed by a finger from outside the body.
